# Galangin Reverses H_2_O_2_-Induced Dermal Fibroblast Senescence via SIRT1-PGC-1α/Nrf2 Signaling

**DOI:** 10.3390/ijms23031387

**Published:** 2022-01-26

**Authors:** Jian-Jr Lee, Shang-Chuan Ng, Jia-Yun Hsu, Hsun Liu, Chih-Jung Chen, Chih-Yang Huang, Wei-Wen Kuo

**Affiliations:** 1 Department of Plastic and Reconstructive Surgery, China Medical University Hospital, Taichung 404, Taiwan; D33977@mail.cmuh.org.tw; 2 School of Medicine, China Medical University, Taichung 404, Taiwan; 3 Department of Biological Science and Technology, College of Life Sciences, China Medical University, Taichung 404, Taiwan; u106078704@cmu.edu.tw (S.-C.N.); jiayun@gmail.com (J.-Y.H.); liuxun@gmail.com (H.L.); 4 Ph.D. Program for Biotechnology Industry, China Medical University, Taichung 404, Taiwan; 5 Division of Breast Surgery, Department of Surgery, China Medical University Hospital, Taichung 404, Taiwan; d10778@mail.cmuh.org.tw; 6 Cardiovascular and Mitochondrial Related Disease Research Center, Hualien Tzu Chi Hospital, Buddhist Tzu Chi Medical Foundation, Hualien 970, Taiwan; cyhuang@cmu.edu.tw; 7 Center of General Education, Buddhist Tzu Chi Medical Foundation, Tzu Chi University of Science and Technology, Hualien 970, Taiwan; 8 Department of Medical Research, China Medical University Hospital, China Medical University, Taichung 404, Taiwan; 9 Graduate Institute of Biomedical Sciences, China Medical University, Taichung 404, Taiwan; 10 Department of Medical Laboratory Science and Biotechnology, Asia University, Taichung 404, Taiwan

**Keywords:** SIRT1, PGC-1α, UVB, senescence, HS68 human dermal fibroblast cells

## Abstract

UV radiation and H_2_O_2_ are the primary factors that cause skin aging. Both trigger oxidative stress and cellular aging. It has been reported that deacetylase silent information regulator 1 (SIRT1), a longevity gene, enhances activation of NF-E2-related factor-2 (Nrf2)*,* as well as its downstream key antioxidant gene hemeoxygenase-1 (HO-1), to protect cells against oxidative damage by deacetylating the transcription coactivator PPARγ coactivator-1α (PGC-1α). Galangin, a flavonoid, possesses anti-oxidative and anti-inflammatory potential. In the present study, we applied Ultraviolet B/H_2_O_2_-induced human dermal fibroblast damage as an in vitro model and UVB-induced photoaging of C57BL/6J nude mice as an in vivo model to investigate the underlying dermo-protective mechanisms of galangin. Our results indicated that galangin treatment attenuates H_2_O_2_/UVB-induced cell viability reduction, dermal aging, and SIRT1/PGC-1α/Nrf2 signaling activation. Furthermore, galangin treatment enhanced Nrf2 activation and nuclear accumulation, in addition to inhibiting Nrf2 degradation. Interestingly, upregulation of antioxidant response element luciferase activity following galangin treatment indicated the transcriptional activation of Nrf2. However, knockdown of SIRT1, PGC-1α, or Nrf2 by siRNA reversed the antioxidant and anti-aging effects of galangin. In vivo evidence further showed that galangin treatment, at doses of 12 and 24 mg/kg on the dorsal skin cells of nude mice resulted in considerably reduced UVB-induced epidermal hyperplasia and skin senescence, and promoted SIRT1/PGC-1α/Nrf2 signaling. Furthermore, enhanced nuclear localization of Nrf2 was observed in galangin-treated mice following UVB irradiation. In conclusion, our data indicated that galangin exerts anti-photoaging and antioxidant effects by promoting SIRT1/PGC-1α/Nrf2 signaling. Therefore, galangin is a potentially promising agent for cosmetic skin care products against UV-induced skin aging.

## 1. Introduction

Skin senescence is a progressive and complicated biological process, which is regulated by both intrinsic and extrinsic factors, leading to cumulative structural and physiological changes in skin layer and appearance [1]. Intrinsic aging tends to intensify with advancing age, whereas extrinsic aging is primarily caused by exposure to UV irradiation from the sun [2]. Both these factors cause the accumulation of reactive oxygen species, which in turn leads to structural damage to proteins and DNA [3]. In addition, senescent cells also exhibit a robust inflammatory response, reduction in antioxidant-related gene expression, and higher secretion of matrix metalloproteinases (MMPs), which enable the degradation of the extracellular matrix (ECM) in the dermal skin layer [4].

Previous studies have shown that ROS accumulation modulates the antioxidant-related gene hemeoxygenase-1 (HO-1) and activates transcription factor NF-E2-related factor-2 (Nrf2) [5,6]. Under conditions of stress, these antioxidant genes are upregulated and exert their cellular protective function to eliminate excessive ROS production [7,8]. Upon stimulation, Nrf2 nuclear transactivation promotes the Nrf2/antioxidant response element [9] signaling pathway [10]. HO-1, a redox-sensitive inducible protein encoded by ARE promoter regulatory elements, protects dermal fibroblast cells against stress-induced oxidative damage [11]. Therefore, enhancement of antioxidant capacity in the skin by natural compounds may serve as a promising strategy for treating stress-induced oxidative damage.

In addition to these antioxidant-related proteins, deacetylase silent information regulator 1 (Sirtuin 1), an NAD (+)-dependent histone deacetylase, plays a vital role in regulating various biological processes, including cellular senescence, as well as antioxidant, anti-apoptotic, and anti-inflammatory signaling [12]. Sirt1 deacetylates the lysine residues of these transcription factors, such as tumor protein P53 (p53), Forkhead box protein O1, and PPARγ co-activator-1α (PGC-1α), to protect cells against damage [13]. Downstream of Sirt1, deacetylated PGC-1α in turn facilitates the upregulation of Nrf2 and antioxidant-related genes [14]. In contrast, PGC-1α enhances NRF1/TFAM signaling to promote mitochondrial biogenesis [15].

Galangin (3,5,7-trihydroxyflavone), a flavanol found in high concentrations in Alpinia officinarum and other members of the ginger family, that is used in traditional Chinese medicine, has been previously demonstrated to exert anti-cancer, anti-inflammatory, and anti-oxidative effects [16,17,18]. Cumulative evidence shows that galangin executes a novel function in skin therapy, such as in the treatment of allergic inflammation and atopic dermatitis-like skin lesions [19,20]. Our previous studies demonstrated that galangin treatment inhibited H_2_O_2_-induced suppression of Insulin Like Growth Factor-1 (IGF1)/Extracellular Signal-Regulated Kinase (ERK) collagen synthesis signaling in human dermal fibroblasts [21,22]. However, the detailed molecular mechanism by which galangin exerts its anti-oxidative properties on dermal fibroblast cells remains unknown.

## 2. Materials and Methods

### 2.1. Cell Culture, UVB-Irradiation, H_2_O_2_ and Galangin Treatment

HS68 cells were obtained from the Bioresource Collection and Research Center (BCRC, Hsinchu, Taiwan). HS68 cells were subcultured in high-glucose Dulbecco’s modified Eagle medium (DMEM) (Sigma–Aldrich D5796, St. Louis, MO, USA) supplemented with 10% fetal bovine serum (FBS) (HyClone) and 1% penicillin–streptomycin at 37 °C in a humidified incubator under a 5% CO_2_ atmosphere. In H_2_O_2_-exposed cells, HS68 cells were treated with 200 μM H_2_O_2_ for 1 h and treated with galangin for another 23 h. For UVB-exposed cells, the cells were washed with PBS and exposed to the crosslinker containing UVB bulbs at an intensity of 40 mJ/cm^2^. After UVB exposure, cells were treated with galangin for 24 h. Low passage (<20) HS68 cells were used in this study.

### 2.2. Antibodies and Reagents

All the antibodies employed in the present study are as follows: anti-β-gal (sc-19119, Santa Cruz, CA, USA), anti-GAPDH (sc-32233, Santa Cruz, CA, USA), anti-HDAC1 (c-19) (sc-6298, Santa Cruz, CA, USA), anti-HO-1 (H-105) (sc-10 789, Santa Cruz, CA, USA), anti-Nrf2 (ab89443, Abcam, Cambridge, UK), anti-p-Nrf2 (S40) (ab76026, Abcam), anti-PGC-1α (D-5) (sc-518025, Santa Cruz, CA, USA), anti-P16^INK4A^ (10883-1-AP, Proteintech, Manchester, UK), anti-p21 (F-5) (sc-6246, Santa Cruz, CA, USA), and anti-p53 (1C12) (no. 2524, Cell Signaling, Danvers, MA, USA), anti-SIRT1 (B-7) (sc-74465, Santa Cruz, CA, USA), and anti-β-actin (sc-47 778, Santa Cruz, CA, USA). The secondary antibodies, namely anti-rabbit, anti-mouse, and anti-goat, were purchased from Santa Cruz, California. H_2_O_2_, galangin, and resveratrol were purchased from Sigma-Aldrich (St. Louis, MO, USA).

### 2.3. MTT Assay

The effect of cellular cytotoxicity on cell survival was assessed using the MTT assay. Briefly, HS68 cells were seeded in 24 wells. After 24 h, cells were treated with different concentrations of H_2_O_2_, UVB, and galangin. After treatment, MTT solution (5 mg/mL) was added to each well and incubation was carried out for 3 h at 37 °C. The supernatant was removed, and DMSO was added over a 10-min period on a shaker. Cell viability was measured at an absorbance of 570 nm using a spectrophotometer reader (BioTek, Winooski, VT, USA). The experiments were repeated thrice to obtain accurate and sufficient data for statistical analysis.

### 2.4. Western Blotting Analysis and Immunoprecipitation

Proteins were extracted using radioimmunoprecipitation assay (RIPA) buffer (R0278, Sigma–Aldrich, St. Louis, MO, USA) and quantified using the Bradford method. Different percentages of SDS-PAGE (6–12%) were used to isolate different molecular weights of the protein. After isolation, the protein on the gel was transferred to a PVDF membrane (GE Healthcare, Amersham, UK), which was activated using methanol. The transferred membranes were treated with blocking buffer and incubated with specific primary HRP-conjugated antibodies overnight at 4 °C on an orbital shaker. The next day, the membranes were incubated with the corresponding secondary antibodies at room temperature for 1 h. The membranes were photographed using an ImageQuant LAS4000 mini with a chemiluminescence substrate. Further experimental details were performed as described previously [23,24].

The PureProteome™ Protein G Magnetic Bead System (Millipore, MA, USA) was used for immunoprecipitation, according to the manufacturer’s instructions [25]. First, 500 μg of cell lysate was incubated with 2 μg of specific primary antibody, and the mixture was incubated overnight on a rotator at 4 °C. The immunoprecipitated protein was eluted from the magnetic beads at 95 °C for 5 min and then separated by SDS-PAGE.

### 2.5. Luciferase Reporter Assay and siRNA Transient Transfection

The pGL3-ARE plasmid was provided by Being-Sun Wung (National Chiayi University, Chiayi City, Taiwan). The dual luciferase reporter gene (DLR) analysis system (Promega, CA, USA) was used to measure ARE transcription activity. Cells were transfected with the pGL3-ARE luciferase reporter gene construct using PureFection transfection reagent. After a 4-h transfection period, the medium was changed and treatment with different concentrations of galangin was carried out for 24 h. Luciferase activity was measured using a reporter microplate luminometer (Turner Biosystems, Sunnyvale, CA, USA). Sirt1-siRNA (SASI_Hs01_00153666) and PGC-1α-siRNA (SASI_Hs01_00063323) were purchased from Sigma–Aldrich (St. Louis, MO, USA).

### 2.6. SA-β-Gal Staining

An SA-β-gal staining kit (No. 9860, Cell Signaling, Danvers, MA, USA) was used to detect senescent cells. HS68 fibroblasts were seeded on slides for 24 h and then subjected to different treatment procedures. The cells were fixed with 4% paraformaldehyde at room temperature for 10 min and washed twice with ice-cold PBS. The cells were then incubated with staining solution overnight at 37 °C. Images were captured using a light microscope (Olympus DP73, Tokyo, Japan).

### 2.7. Nuclear and Cytoplasmic Separation

A nuclear and cytosol separating kit (BioVision, Milpitas, CA, USA) was used to isolate the nuclear and cytosolic proteins, according to the manufacturer’s instructions [26]. Nuclear fractions were resuspended in CEB-A mix (proteinase inhibitor cocktail, DTT), and cytoplasmic fractions were resuspended in NEB mix (proteinase inhibitor cocktail, DTT). Nuclear and cytosolic proteins were separated by SDS-PAGE.

### 2.8. Immunofluorescence Staining

HS68 cells were fixed with 4% paraformaldehyde and permeabilized with 0.1% Triton X-100 for 15 min at room temperature before staining with the primary antibody p-Nrf2 (S40) (ab76026; Abcam, Cambridge, UK). Incubation with Alexa 488 goat anti-rabbit IgG secondary antibodies (A11008, Invitrogen, Carlsbad, CA, USA) was carried out for 2 h at room temperature. Staining with 4′,6-diamidino-2-phenylindole (DAPI) was performed to ensure localization of the nucleus. Images were captured using a fluorescence microscope (Olympus DP73, Tokyo, Japan).

### 2.9. Experimental Animals and Treatment Protocols

C57BL/6J nude mice were kept at a constant temperature (22 °C), with a light/dark cycle of 12 h, and were provided access to food and tap water. Briefly, dorsal skin sections from five-week-old mice were subdivided into the following four areas: (i) control group + vehicle; (ii) UVB-irradiation + vehicle group; (iii) UVB irradiation + low-dose galangin (12 mg/kg) group; and (iv) UVB irradiation + high-dose galangin (24 mg/kg) group. The dorsal skin areas were topically applied with vehicle (propylene glycol: ethanol: H_2_O = 1:1:8) or galangin (100 μL each) and were irradiated with UVB (150 mJ/cm^2^ per exposure, 3 times a week for 8 weeks). Each group consisted of six animals. All animals were weighed and inhaled with 1–3% isoflurane before sacrificed.

### 2.10. Immunohistochemistry

The slides were immunostained with anti-PGC-1α, anti-Sirt1, and anti-β-gal antibodies using an UltraVision LP detection system (Vector Laboratories, Burlingame, CA, USA), according to the manufacturer’s instructions [27]. Tissue biopsies were dried overnight at 60 °C. The following day, tissues were deparaffinized in xylene for 40 min and rehydrated with a graded series of ethanol. The slides were covered with permanent mounting medium (Sigma–Aldrich, St. Louis, MO, USA) and photographed under a microscope (Olympus DP73, Tokyo, Japan).

### 2.11. Statistical Analysis

Statistical analysis was performed using Graph Pad Prism5 statistical software (San Diego, CA, USA). The overall significant difference between the averages of multiple groups was evaluated using the analysis of variance. The data were analyzed using a separate one-way analysis of variance. Significant differences between individual means were determined using Tukey’s test. All results were quantified using ImageJ software.

## 3. Results

### 3.1. Galangin Inhibited UVB- and H_2_O_2_-Induced Proliferation Reduction in HS68 Cells

**Figure 1:**
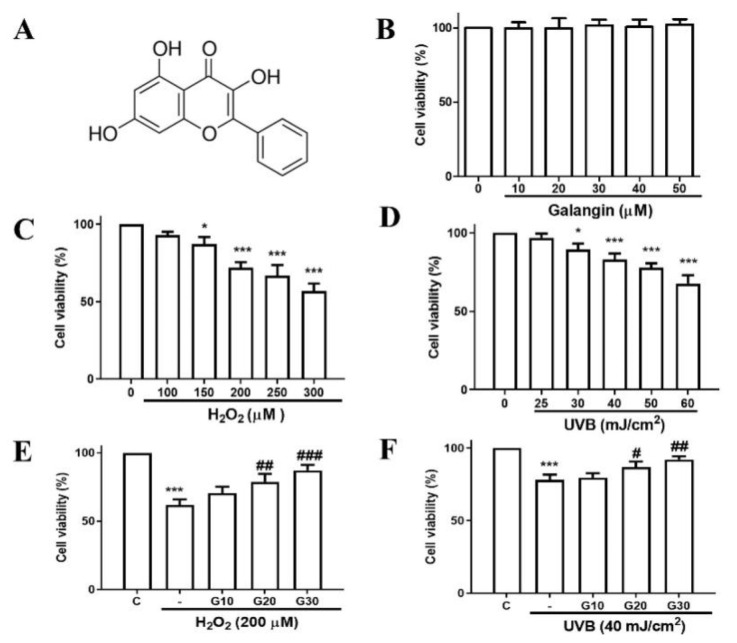
Galangin inhibits UVB- and H_2_O_2_-induced proliferation reduction in HS68 cells. (**A**) Structure of galangin (3,5,7-trihydroxyflavone). (**B**–**D**) HS68 cells were treated with galangin, H_2_O_2_, and UVB, at the indicated concentrations/intensities for 24 h. (**E**,**F**) HS68 cells were exposed to H_2_O_2_ (200 μM) or UVB (40 mJ/cm^2^) and then co-treated with galangin at various concentrations (10–30 μM). The MTT assay was used to determine cell viability. Values shown are means ± SE. Quantification of the results is shown (*n* = 3) * *p* < 0.05, *** *p* < 0.001 vs. control cells; ^#^ *p* < 0.05, ^##^ *p* < 0.01, ^###^ *p* < 0.001 vs. H_2_O_2_- or UVB-treated cells.

To investigate the cellular cytotoxicity of galangin (Figure 1A) in vitro, HS68 cells were treated with different doses of galangin (0, 10, 20, 30, 40, and 50 μM) for 24 h. The MTT assay results showed that galangin was not cytotoxic to HS68 cells (Figure 1B). Next, we determined whether H_2_O_2_ or UVB induces cell-proliferation reduction in HS68 cells. The cells were exposed to different doses of H_2_O_2_ (100, 150, 200, 250, and 300 μM) and UVB (25, 30, 40, 50, and 60 mJ/cm^2^) for 24 h. The results indicated that cell viability decreased in a dose-dependent manner following H_2_O_2_/UVB exposure (Figure 1C,D). Next, we examined the protective effects of galangin in H_2_O_2_- and UVB-exposed cells. The results indicated that galangin was able to protect HS68 cells against H_2_O_2_- and UVB-induced dermal cell death, and a dose of 30 μM was chosen for subsequent experiments (Figure 1E,F).

### 3.2. Effect of Galangin on the SIRT1/PGC-1α/Nrf2 Pathway and Upregulation of Antioxidant Genes (HO-1) in HS68 Cells Exposed to UVB/H_2_O_2_

**Figure 2:**
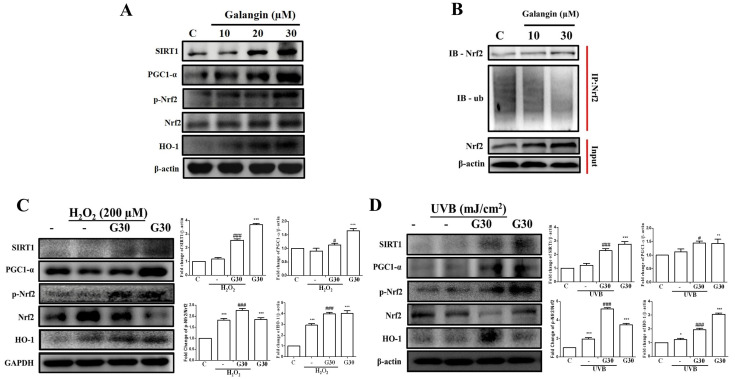
Effects of galangin on the Sirt1/PGC-1α/Nrf2 pathway and upregulation of antioxidant genes (HO-1) in HS68 cells exposed to UVB/H_2_O_2_. (**A**) HS68 cells were treated with different concentrations of galangin for 24 h. (**B**) The binding of Ub with Nrf2 was detected using immunoprecipitation under different concentrations of galangin for 24 h. (**C**,**D**) HS68 cells were exposed to H_2_O_2_ (200 μM) or UVB (40 mJ/cm^2^) and then co-treated with galangin at a specific concentration (30 μM). Protein levels of Sirt1, PGC-1α, Nrf2, p-Nrf2, and HO-1 were detected by Western blot. Values shown are means ± SE. Quantification of the results is shown (*n* = 3) * *p* < 0.05, ** *p* < 0.01, *** *p* < 0.001 vs. untreated control cells; ^#^ *p* < 0.05, ^###^ *p* < 0.001 vs. H_2_O_2_/UVB-treated cells.

To evaluate the involvement of the SIRT1/PGC-1α/Nrf2 pathway in the antioxidant effect of galangin against UVB/H_2_O_2_-induced dermal fibroblast oxidative damage, Western blotting was performed to detect the protein expression of SIRT1, PGC-1α, p-Nrf2, Nrf2, and HO-1. Galangin treatment dose-dependently increased the protein levels in SIRT-1, PGC1-α, p-Nrf-2, and HO-1 (Figure 2A). Immunoprecipitation experiments further confirmed that galangin treatment prevents Nrf-2 degradation through ubiquitination (Figure 2B). Furthermore, treatment with galangin in UVB- and H_2_O_2_-exposed cells substantially induced Sirt-1/PGC-1α/Nrf-2 signaling (Figure 2C,D). These results indicated that galangin ameliorates H_2_O_2_- and UVB-induced oxidative damage in dermal fibroblasts by enhancing the Sirt-1/PGC-1α/Nrf-2 signaling pathway.

### 3.3. Galangin Triggered Nrf2 Nuclear Translocation and ARE Transcriptional Activation in HS68 Cells Exposed to H_2_O_2_

**Figure 3:**
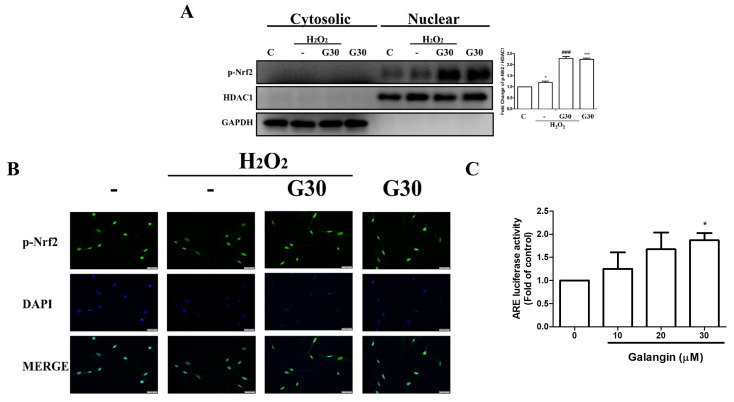
Galangin triggers Nrf2 nuclear translocation and ARE transcriptional activation in HS68 cells exposed to H_2_O_2_. (**A**,**B**) HS68 cells were exposed to H_2_O_2_ (200 μM) or UVB (40 mJ/cm^2^) and then co-treated with galangin at a specific concentration (30 μM). p-Nrf2 expression was estimated in the cytosolic and nuclear fractions. The p-Nrf2 protein level was detected by Western blot. (**C**) p-Nrf2 nuclear translocation was further ascertained by immunofluorescence staining. An anti-Nrf2 antibody and a FITC-conjugated second antibody were used to detect p-Nrf2 cellular distribution. DAPI staining indicated the nucleus location (blue). The images were acquired using florescence microscopy (200×). HS68 cells were transfected with ARE-luciferase construct for 24 h. The cells were then treated with different doses of galangin (10, 20, and 30 µM) for 24 h and assayed for luciferase activity. Values shown are means ± SE. Quantification of the results is shown (*n* = 3) * *p* < 0.05, *** *p* < 0.001 vs. untreated control cells; ^###^ *p* < 0.001 vs. H_2_O_2_-treated cells.

Nrf2/ARE activation has been reported to regulate antioxidant genes. Next, we examined the nuclear translocation pattern of Nrf-2 and its transcriptional activation. Our Western blot analysis results showed that nuclear accumulation of p-Nrf-2 in H_2_O_2_-induced HS68 cells was further enhanced by galangin treatment (Figure 3A). Furthermore, immunofluorescence images of p-Nrf2 nuclear co-localization showed similar results (Figure 3B). Next, we measured the ARE activity level upon galangin treatment, and our results showed that Nrf-2-dependent luciferase activity was markedly increased in a dose-dependent manner (Figure 3C). In conclusion, galangin considerably augmented the nuclear translocation and transcriptional activation of Nrf-2, which is a transcription factor that regulates the production of antioxidant proteins to protect dermal fibroblast cells against oxidative damage.

### 3.4. Galangin, as Well as Resveratrol (Sirt1 Activator), Enhanced the Sirt1/PGC-1α/Nrf2 Pathway and Antioxidant Genes (HO-1) in HS68 Cells following UVB/H_2_O_2_-Induced Damage

**Figure 4:**
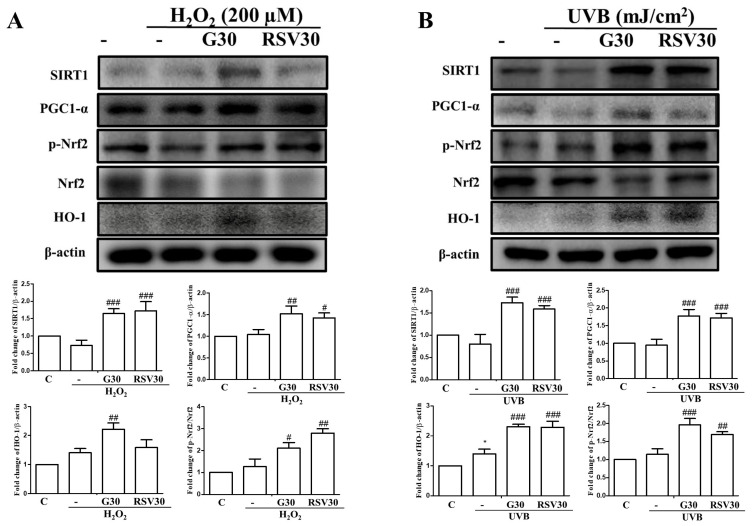
Galangin enhances the Sirt1/PGC-1α/Nrf2 pathway and its downstream gene (HO-1), as well as resveratrol (Sirt1 activator), in HS68 cells under UVB/H_2_O_2_-induced damage. (**A**,**B**) HS68 cells were exposed to H_2_O_2_ (200 μM) or UVB (40 mJ/cm^2^) and then co-treated with galangin (30 μM) or resveratrol (30 µM) as indicated in related graphs. The protein levels of Sirt1, PGC-1α, p-Nrf2, Nrf2, and HO-1 were detected by Western blot. Values shown are means ± SE. Quantification of the results is shown (*n* = 3) * *p* < 0.05 vs. untreated control cells; ^#^ *p* < 0.05, ^##^ *p* < 0.01, ^###^ *p* < 0.001 vs. H_2_O_2_/UVB-treated cells.

Previous study showed that resveratrol was capable of activating Sirt1 signaling and protecting cells against oxidative damage [28,29]. Herein, we compared the protective effects of galangin and resveratrol at the same dose (30 μM) on UVB/H_2_O_2_-exposed cells. Interestingly, galangin-treated cells exhibited similar levels of enhancement in Sirt-1/PGC-1α/Nrf-2 signaling compared with cells treated with resveratrol at the same dose of 30 μM (Figure 4A,B). Our results demonstrated that galangin could act as another Sirt-1-activating compound.

### 3.5. Anti-Aging Effects of Galangin on UVB/H_2_O_2_-Induced HS68 Cell Senescence

**Figure 5:**
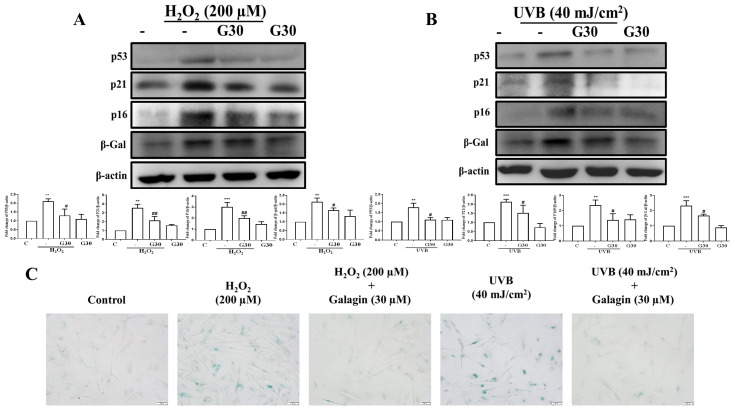
Anti-aging effects of galangin on UVB/H_2_O_2_-induced senescence of HS68 cells. (**A**,**B**) HS68 cells were exposed to H_2_O_2_ (200 μM) or UVB (40 mJ/cm^2^) and then co-treated with galangin at a specific concentration (30 μM). Protein levels of p53, p21, p16, and β-Gal were detected by Western blot. (**C**) After treatment, cells were stained using an SA-β-Gal staining kit to detect SA-β-Gal activity. Positive cells were stained in green color, which indicated senescent cells. Values shown are means ± SE. Quantification of the results is shown (*n* = 3) ** *p* < 0.01, *** *p* < 0.001 vs. untreated control cells; ^#^ *p* < 0.05, ^##^ *p* < 0.01, vs. H_2_O_2_/UVB-treated cells.

To identify whether exposure to UVB or H_2_O_2_ causes dermal aging, Western blot analysis was performed to investigate the anti-aging effect of galangin. Our results demonstrated that aging markers, such as p53, p21, and p16, were upregulated following exposure to H_2_O_2_ (200 μM) or UVB (40 mJ/cm^2^); moreover, this result was reversed by galangin treatment (Figure 5A,B). SA-β-gal staining was employed to detect the aged dermal fibroblasts. Our results indicated that UVB or H_2_O_2_ exposure increased the number of SA-β-gal-positive cells. However, treatment with galangin reversed UVB/H_2_O_2_-induced dermal fibroblast cell senescence (Figure 5C).

### 3.6. Silencing of Sirt1 or PGC-1α by siRNA Diminished the Protective Effects of Galangin under H_2_O_2_ Exposure in HS68 Cells

**Figure 6:**
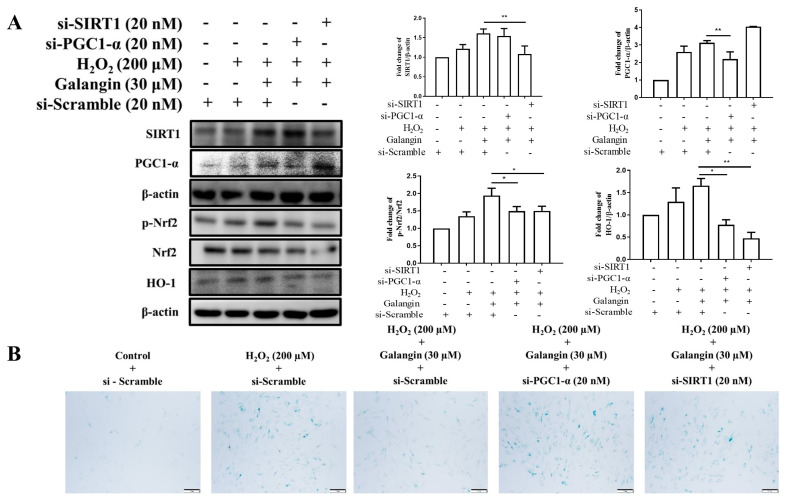
Silencing of *Sirt1* or *PGC-1α* diminishes the protective effects of galangin under H_2_O_2_ exposure in HS68 cells. (**A**) Levels of Nrf2, p-Nrf2, and HO-1 measured by Western blot in response to *Sirt1* and *PGC-1α* silencing. (**B**) After treatment, cells were stained using an SA-β-Gal staining kit to detect SA-β-Gal activity. Positive cells were stained in green, which indicates senescent cells. Values shown are means ± SE. Quantification of the results is shown (*n* = 3) * *p* < 0.05, ** *p* < 0.01, vs. galangin plus H_2_O_2_-treated cells.

To ensure that galangin activates Sirt1/PGC-1α/Nrf2 signaling to attenuate skin aging in H_2_O_2_-exposed cells, we used siRNA to silence Sirt1 and PGC-1α expression; this would enable identification of the protective effects of galangin. Our data showed that silencing Sirt1 or PGC-1α effectively hindered Nfr2 activation and HO-1 protein expression (Figure 6A). Furthermore, galangin attenuated H_2_O_2_-induced upregulation of SA-β-gal-positive cells, which was reversed following the knockdown of Sirt1 or PGC-1α (Figure 6B). These results indicated that the induction of Nrf2 in attenuating aging is mediated via Sirt1 and PGC-1α signaling in dermal fibroblast cells exposed to H_2_O_2_.

### 3.7. Silencing of Nrf2 by siRNA Diminished the Protective Effects of Galangin under H_2_O_2_ Exposure in HS68 Cells

**Figure 7:**
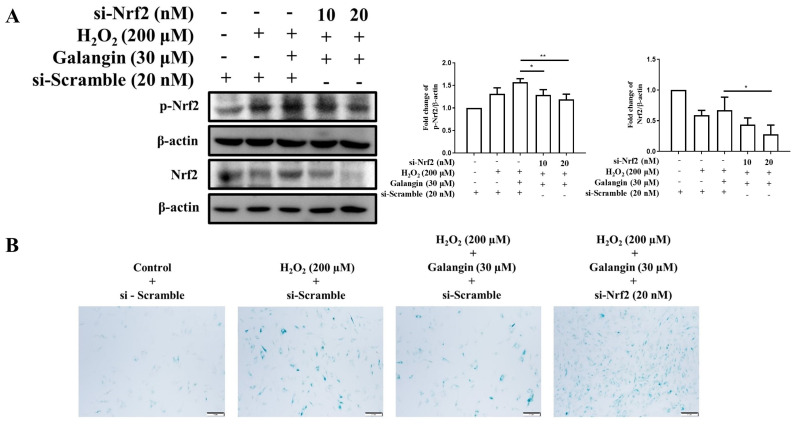
Silencing of *Nrf2* diminishes the protective effects of galangin under H_2_O_2_ exposure in HS68 cells. (**A**) p-Nrf (active) and Nrf protein levels were detected by Western blotting in response to siRNA silencing to ensure knockdown efficiency. β-actin acted as loading control. (**B**) After treatment, cells were stained using an SA-β-Gal staining kit to detect SA-β-Gal activity. Positive cells were stained in green, which indicates senescent cells. Values shown are means ± SE. Quantification of the results is shown (*n* = 3) * *p* < 0.05, ** *p* < 0.01, vs. galangin plus H_2_O_2_-treated cells.

To demonstrate that the protective effects of galangin are mediated by Nfr2 activation, siRNA-Nrf2 transfection was used to silence Nfr2 expression. Successful knockdown of Nrf2 was confirmed by Western blot analysis (Figure 7A). Furthermore, galangin attenuated H_2_O_2_-induced upregulation of SA-β-gal-positive cells, which was reversed following Nrf2 knockdown (Figure 7B). These findings confirmed that the anti-aging effect of galangin is mediated through Nrf2 activation.

### 3.8. Galangin Alleviated UVB-Induced Skin Photodamage in C57BL/6J Nude Mice

**Figure 8:**
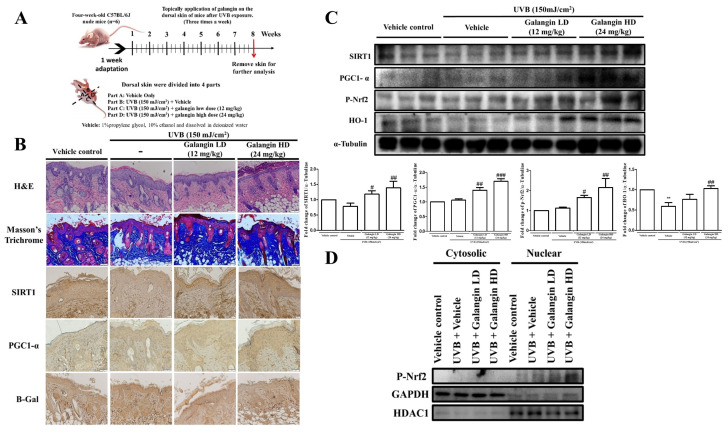
Galangin alleviates UVB-induced skin photodamage in C57BL/6J nude mice. (**A**) The experimental animal protocol has been described in detail in the Materials and Methods section. (**B**) Skin morphology and collagen levels were determined by H&E stain and Masson’s trichrome stain, respectively. Sirt1, PGC1-α, and β-Gal were detected by immunohistochemistry. (**C**) Western blot analysis of SIRT1, PGC1-α, p-Nrf, and HO-1 protein levels in the dorsal skin of the mice. α-Tubulin acted as a loading control. (**D**) p-Nrf2 expression in the cytosol and nucleus was analyzed using a nucleus and cytosol extraction kit and detected by Western blotting. Values shown are means ± SE. Quantification of the results is shown (*n* = 3) ** *p* < 0.01 vs. vehicle control group; ^#^ *p* < 0.05, ^##^ *p* < 0.01, ^###^ *p* < 0.001 vs. UVB-irradiated group.

**Figure 9:**
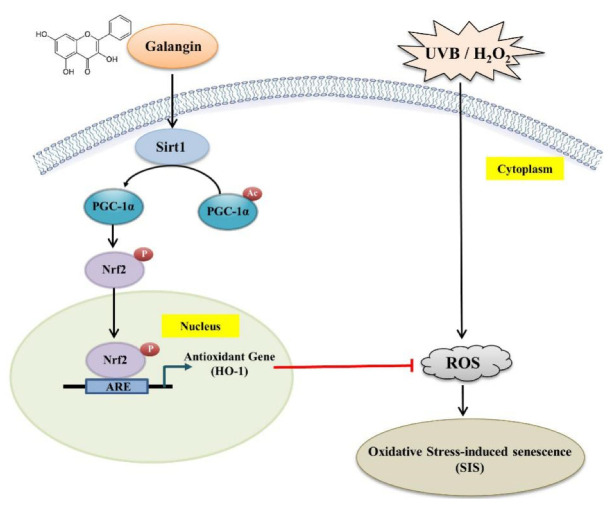
Scheme summarizing the inhibition of H_2_O_2_-induced skin oxidative damage by galangin via the upregulation of antioxidant genes (*HO-1*) through the Sirt1/PGC-1α/Nrf2 pathway in HS68 human dermal fibroblast cells. Sirt 1: silent information regulator 1; PGC-1α: PPARγ coactivator-1α; Nrf2: NF-E2-related factor-2; HO-1: hemeoxygenase-1.

The protective effects of galangin in vivo were demonstrated by the topical administration of galangin on the dorsal skin of 4-week-old C57BL/6J nude mice following UVB (150 mJ/cm^2^) exposure three times a week over a duration of 8 weeks (Figure 8A). The skin epidermis thickness, collagen content, as well as protein levels of Sirt1, PGC-1α, Nfr2, HO-1, and β-Gal were examined. Our data showed that topical application of galangin on UVB-irradiated dorsal skin of mice led to a dose-dependent decrease in epidemic thickness and an enhancement of collagen fiber density in the dermis (Figure 8B). The immunohistochemistry (IHC) images of the obtained results showed a remarkable upregulation in the Sirt1 and PC-1α levels in galangin-treated mice following UVB irradiation (Figure 8B). Moreover, the β-Gal levels, which are an indicator of senescence, were reduced upon galangin treatment (Figure 8B). Furthermore, Western blot analysis of the skin tissue showed that the protein levels of Sirt1, PGC-1α, p-Nrf2, and HO-1, as well as the nuclear transactivation of Nrf-2, were dose-dependently increased in the galangin-treated groups compared with the corresponding parameters in the UVB-irradiated groups (Figure 8C,D). In conclusion, our study demonstrated that galangin protected human dermal fibroblasts against H_2_O_2_- or UVB-induced damage through enhanced Sirt1/PGC-1α/Nrf2 signaling, as well as its downstream effector HO-1, to attenuate oxidative stress-induced senescence (Figure 9).

## 4. Discussion

Human skin senescence is a progressive process resulting from ROS accumulation, followed by human fibroblast dysfunction and wrinkle formation. Skin senescence is divided into two major types: intrinsic aging and extrinsic aging. Both are strongly linked to increased generation of ROS, which is an imbalance between free radical production and anti-oxidative defense in the skin [30]. In the present study, we utilized two in vitro models to induce human dermal fibroblast damage, including H_2_O_2_ and UVB, to mimic the intrinsic and extrinsic effects of skin aging, respectively. Additionally, UVB-irradiated dorsal skin of nude mice was used as a skin photoaging model in the in vivo study. In the present study, we found that treatment with galangin, a natural flavonoid derived from *Alpinia officinarum*, considerably alleviates the deleterious effects induced in both in vitro and in vivo models. The protective properties of galangin were accomplished with the upregulation of Sirt1 and PGC-1α, which in turn augmented the expression of antioxidant genes, such as HO-1, and reduced dermal senescence. The antioxidant responses of galangin are activated by Nrf2/ARE signaling. In addition, galangin-induced Nrf2 activation was found to be regulated by the Sirt1 and PGC-1α signaling pathways. This was further confirmed by silencing Sirt1 and PGC-1α using siRNA, which attenuated Nrf2 activation and enhanced cellular senescence. Further evidence showed that Nrf2 knockdown cells were unable to rescue the anti-aging effects of galangin; consequently, it was inferred that Nrf2 activation is essential for the action of galangin in reviving stress-induced dermal fibroblast aging. In summary, our findings indicated that galangin substantially reverses H_2_O_2_-induced cellular senescence by promoting SIRT1/PGC-1α/Nrf2 signaling.

Antioxidants play an essential role in protecting cells against oxidative stress and damage by eliminating excessive free radicals [31]. Galangin is a flavonoid compound with anti-oxidative potential, serving as a source of readily available hydrogen to form its 3,5,7-trihydroxyl group, which scavenges free radicals [32]. The antioxidant potential of the therapeutic effects of galangin has been reported in various diseases, including diabetes mellitus and nephrotoxicity [31,33]. Furthermore, a study demonstrated that galangin restores hepatic mitochondrial function by improving the antioxidant status, which potentially decreases the peroxidation of membrane lipids [18]. Zhang et al. reported that galangin is primarily driven by hydrophobic interactions and hydrogen bonds within the active site of α-glucosidase, which moderately inhibits α-glucosidase in the treatment of type 2 diabetes [34]. Most importantly, our recent study indicated that galangin treatment alleviated H_2_O_2_/UVB-induced intracellular and mitochondrial ROS production as well as mitochondria membrane potential (MMP) imbalance [35]. In addition, another study showed that galangin could promote Nrf2 signaling to protect keratinocytes cells from UVB-induced damage [36]. Sirt1 and Nrf2 signaling were enhanced by galangin to reduce oxidative stress in cadmium-induced renal damage [37]. Therefore, we assumed that the protective effect of galangin against H_2_O_2_/UVB-induced damage is mediated by its antioxidant efficacy. In our prior studies, we reported that galangin protects skin cells against ROS-induced aging and alleviates oxidative stress and inflammatory responses in HS68 cells. It was also identified that galangin treatment attenuated the levels of phosphorylated NF-κB and pro-inflammatory cytokines, which were increased by H_2_O_2_. The effect of galangin probably was induced by enhancing the IGF1R/Akt survival pathways, which then led to an anti-inflammatory effect [21,22].

Flavonoids, including luteolin, quercetin, apigenin, and naringenin, were previously shown to enhance Sirt1 expression or activate Sirt1 [38,39,40,41]. However, relatively few studies have reported that galangin regulates Sirt1 expression. A previous study indicated that galangin is capable of upregulating Sirt1 and deacetylated LC3, which in turn induces hepatocellular carcinoma autophagy and apoptosis [42]. Sirt1 plays multiple roles in cancer and age-related diseases [43]. Shen et al. reported that fullerenol activates Sirt1, leading to decreased levels of acetyl-p53 and p21/Waf1/Cip1, in protecting retinal pigment epithelium (RPE) cells against senescence under ROS stimulation [44]. Additionally, Nrf2 is not only responsible for regulating antioxidant, anti-inflammatory, and detoxifying proteins but is also an important modulator of cellular longevity. Nrf2 is translocated into the nucleus and binds AREs in the promoter region of genes encoding antioxidant enzymes, such as HO-1 [36]. At the molecular level, Nrf2, a cytoprotective transcription factor, was demonstrated to hamper UV-induced skin carcinogenesis and attenuate UVA-induced oxidative damage in skin dermal fibroblasts [45]. Moreover, luteolin has been reported to induce Sirt1/Nrf2 signaling to protect hepatocytes against HgCl_2_-induced ROS generation, inflammatory responses, and cellular apoptosis [46]. Zhang et al. reported that pyrroloquinoline quinine treatment protects against UVA-induced dermal aging by activating the SIRT1/Nrf2/HO-1 pathway [43]. In our study, galangin was capable of activating Sirt1. Furthermore, all these findings are similar to our obtained results, implying that the protective mechanism of galangin may be mediated by the activation of the SIRT1/Nrf2/HO-1 signaling pathway to attenuate H_2_O_2_-induced skin dermal aging.

## 5. Conclusions

In conclusion, our previous study showed that galangin inhibits dermal fibroblast inflammation by enhancing the IGF1R/Akt survival pathway in H_2_O_2_-induced senescence. In the present study, we further explored the anti-aging effects of galangin in attenuating H_2_O_2_-induced senescence by upregulating the Sirt1/PGC-1α/Nrf2 signaling pathway and downregulating specific senescence-associated markers, such as p53, p21, p16, and SA-β-gal. A more detailed mechanism involved in hindering oxidative stress-induced dermal aging by galangin was elucidated in the present study. Therefore, the findings of our study demonstrated that galangin may be applied as a versatile natural candidate in the development of skin care products.
